# From Bench to Insight: Rapid Pathogen Genomic Surveillance Workflow for SARS-CoV-2 and Emerging Pathogens

**DOI:** 10.3390/genes17060632

**Published:** 2026-05-30

**Authors:** Chelsea Zimmer, Selena McVay, Keely Starke, Kimily Hughley, Sara N. Koenig, Venkat Sundar Gadepalli

**Affiliations:** Office of Research, College of Medicine, The Ohio State University Wexner Medical Center, Columbus, OH 43201, USA

**Keywords:** WDL workflow, clade, lineage, S5 ion torrent suite

## Abstract

**Background/Objectives**: Clinical surveillance of infectious diseases caused by viruses, such as SARS-CoV-2, is important for effective intervention and preventing potential epidemics or pandemics. The development of cost-effective whole genome sequencing technologies has facilitated worldwide efforts to sequence viral genomes. The array of sequence data generated across the globe offers diverse opportunities to study SARS-CoV-2 evolutionary dynamics and serves as a foundation for different research questions in the future. Even though bioinformatics tools are rapidly developed for accessing and analyzing large-scale data from public repositories, surveillance labs lack streamlined pipelines to handle high sample volumes and efficiently identify mutations for variant reporting with minimal computational expertise. **Methods**: We have developed a SARS-CoV-2 mutational analysis pipeline using Workflow Description Language (WDL), which is open-source and combines various steps in an analysis workflow with human-readable syntax. Thus, users with minimal informatics background can easily adapt the workflow while creating a local data repository within their institution. The pipeline processes input FASTA files and quality control files from Ion Torrent S5, performs clade and variant assignments, integrates patient metadata, and stores the results into a REDCap database. **Results**: In this framework, REDCap acts as the core data backbone for run-level tracking and result storage. To further enhance the utility of our REDCap-based data capture system, we have developed an intuitive interactive dashboard. This interface seamlessly connects with the REDCap data sources, providing real-time monitoring, interactive visualization, and the ability to create a consolidated variant report. **Conclusions**: Our overall approach streamlines processes in managing complex genomic data and offers easy adaptation to empower other molecular labs.

## 1. Introduction

Zoonotic transmission, a term defining a viral infection affecting humans by viruses that are commonly reported in animals, is far from a rare phenomenon. A comprehensive literature review by Taylor L.H et al. [1] found that of 1415 species of infectious organisms known to be pathogenic to humans, approximately 61% were of animal origin. Diseases such as Brucella, HIV, Salmonella, rabies virus, Ebola, and highly pathogenic avian influenza have been a threat to public health for decades [2,3,4]. These zootonic diseases have caused significant morbidity and mortality, challenging healthcare systems. The increases in these outbreaks represent a significant public health concern. The outbreak of Coronavirus Disease 2019 (COVID-19) was caused by an RNA virus strain from the family Coronaviridae with 89.1% nucleotide similarity to a group of coronaviruses [5]. Unlike other viral transmissions from animals to humans, such as HIV/AIDS, Ebola virus disease, SARS (Severe Acute Respiratory Syndrome), and MERS (Middle East Respiratory Syndrome), the novel coronavirus SARS-CoV-2 was highly contagious, which caused detrimental effects on the human population and economic conditions across the globe [6]. As viral transmission increases, so do opportunities for viral mutation and adaptation. The high transmission rates of SARS-CoV-2 resulted in multiple emerging variants due to mutations in the spike protein. The spike protein helps the virus to bind with human Angiotensin-converting enzyme 2 (ACE2), a primary receptor for host cell entry [7]. The impacts of mutations in the spike protein of SARS-CoV-2 have been well-studied and published. Some of the relevant amino acid substitutions in the spike protein include D614G, N501Y, L452R, and E484K [8]. These mutations have significant functional consequences that result in the virus gaining characteristics. The first notable mutation is the D614G substitution found in the B.1 lineage, from which all SARS-CoV-2 variants of concern (VOC) have emerged [9]. During the spread in 2021, VOCs such as alpha, beta, and gamma all shared the N501Y mutation. The N501Y substitution enhances the virus’s binding affinity to the ACE2 receptor, thereby increasing transmissibility [10]. The L452R substitution has replaced earlier mutations, and this variant is characterized by increased spike stability, viral infectivity, and evading the host immune system, thereby promoting viral replication resulting in higher virulence [11,12]. The E484K and K417N/T mutations, present in the beta and gamma variants, were linked to reduced neutralization by antibodies, impacting vaccine efficacy [12,13]. The delta (B.1.617.2) variant that was associated with pathogenicity and transmissibility during March 2021 in India carried a P681R substitution. This mutation occurred in a highly conserved region of the spike protein, thereby resulting in a variant exhibiting enhanced pathogenicity compared to its parent variant [14]. Collectively, these mutations give each variant a unique profile of transmissibility, virulence, and immune evasive functionality. These emerging variants pose a continuous challenge as they can lead to new waves of infection. Therefore, it becomes crucial to monitor the variant’s emergence constantly. It is also important for surveillance studies to provide accurate information, monitor infection trends, and support evidence-based public health interventions [15,16]. The WHO Virus Evolution Working Group has established an easy-to-use definition system for distinguishing these emerging variants that considers the extent to which an outcome may affect public health efforts, such as evasion of immunity or diagnostic testing [17]. Variants of concern (VOC) are designated due to high transmissibility and detrimental outcomes on public health, while Variants Under Monitoring (VUM) are known to cause changes in viral characteristics but without sufficient evidence to determine public health risk [18,19]. This system has organized the scientific community’s efforts to delve into the genomic landscapes of the viral genome.

Historically, next-generation sequencing techniques required complex chemistry and specialized training of laboratory technicians and other specialized personnel. However, with improvements in automation and nanotechnology, these inherently specialized methodologies have been simplified and are easily adapted by the novice user in a clinical molecular lab. Whole genomic sequencing methodologies offer detailed insights to identify viral variants. The rapid development in next-generation sequencing (NGS) techniques, such as amplicon sequencing, offers a step further regarding cost-effectiveness and improved turnaround times for whole genome sequencing. To determine the origins and evolution history of the SARS-CoV-2, researchers have employed the metagenomic NGS (mNGS) approach, which offers an unbiased and hypothesis-free examination of a sample [5,20]. Instead of targeting specific regions of a genome, this approach sequences all nucleic acids available in a sample containing a mixed population of microorganisms. These sequences are later aligned to a wide range of databases that help determine their origins across different taxa or organisms. This laid the foundation for the development and characterization of a reference genome for SARS-CoV-2 that paved the way for rapid whole and targeted genome sequencing [21,22,23]. Our NGS workflow and analysis pipeline has been implemented on genome sequencing data generated using the ThermoFisher Ion AmpliSeq™ SARS-CoV-2 Research Panel (Catalog number: A51305). The panel enables the detection of a low viral titer sample, with two primer pools that target 237 amplicons specific to the SARS-CoV-2 and five human controls. With an amplicon length range of around 125 to 275 bp, the panel provides greater than 99% coverage of the SARS-CoV-2 genome (~30 kb) and a faster turnaround time compared to traditional sequencing methods, particularly those relying on the Illumina MiSeq or other conventional approaches like shotgun metagenomics or earlier amplicon-based protocols [23]. Thus, this approach is an ideal technique for public health surveillance.

While TorrentSuite provides an accurate analysis for assigning clades or identifying mutations, ThermoFisher’s methodologies and tools are proprietary and specifically tailored for their sequencing platform. Moreover, the vendor-controlled system, despite its convenience, lags in adaptation of rapidly developing data resources and tools. Open-source data repositories and bioinformatics tools promote widespread acceptance within the research community. They often result in the formation of large NGS data consortiums. Consequently, the methods used by these public data repositories and tools are regularly validated and updated to ensure researchers have access to the most current information.

Public-domain databases such as GenBank, the European Nucleotide Archive (ENA), and DNA bank are well-known nucleotide sequence database repositories. The Global Initiative on Sharing All Influenza Data (GISAID) database (https://www.gisaid.org/, accessed on 26 May 2026) is one such collaboration that has gained global importance for studying the influenza virus. It has now emerged as a main repository for the SARS-CoV-2 viral genome data. As of 2024 more than 16 million SARS-CoV-2 genome sequences have been submitted to this repository [24]. This curated data repository has empowered diagnostic labs, vaccine manufacturers, and researchers in their efforts to counter and understand the dynamic nature of the SARS-CoV-2 genome and has been a prominent resource in the continuous monitoring of emerging variants. These repositories guide public health measures such as vaccine development and the identification of potential outbreaks. With this vast amount of data, a deeper understanding of the virus’s behavior can be achieved by using various bioinformatics tools, which allow handling gigabytes of data and performing sequence alignment, mutation identification, and creation of phylogenetic trees. Nextclade (3.21.2)and Pangolin(4.4) are widely accepted bioinformatics tools that complement each other for researchers to extract valuable insights and understand the ever-evolving SARS-CoV-2 genomic landscape. Nextclade offers both a command line and an easy-to-use web tool to visualize pathogen evolution and epidemic spread. It offers broader genomic analysis using a reference-based approach in assigning clades or building phylogenetic trees in addition to performing quality checks on FASTA sequences submitted [25]. Pangolin offers an additional standardized naming system based on the Pango nomenclature [26]. It is widely used by researchers and public health agencies worldwide in tracking and understanding the global spread of SARS-CoV-2. The web and command line computation tools offer an easy resource for researchers to assign the Pango lineage by querying multiple SARS-CoV-2 genomes. These tools are indispensable for clinical laboratories focusing on genomic surveillance or extracting usable and actionable insights from the huge volumes of SARS-CoV-2 genome sequencing data [27]. These collaborative efforts offer continuous monitoring of currently circulating variants of interest (VOIs). For example, as per the WHO COVID-19 Dashboard, as of 3 February 2025, several variants with Pango lineages—KP.2, KP.3, KP.3.1.1, JN.1.18, LB.1, XEC, and LP.8.1—are being evaluated for their prevalence and posed risk to public health [28]. This real-time evaluation would not be possible without increasing the representation of various research laboratories across the globe and submitting their findings with consortiums such as GISAD, an initiative that involves public–private partnerships.

In our lab SARS-CoV-2 mutation detection and analysis was a collaborative effort between laboratory and bioinformatic personnel. Laboratory personnel determined the result of the patient sample using TaqPath™ COVID-19 Combo Kits. SARS-CoV-2 surveillance and analysis for positive samples were performed using the Ion Ampliseq protocol and Insight Research Panel (A51305). Our lab selected this panel as it offers genome sequencing of SARS-CoV-2 with high sensitivity, including variants of concern, through Next Generation Sequencing (NGS). Workflow efficiency and accuracy were optimized during the unprecedented public health emergency. Positive SARS-CoV-2 samples were initially categorized upon the number of copies from their N gene Ct values as determined by the TaqPath COVID-19 Combo Kit™ (Thermo Fisher Scientific, Waltham, MA, USA). As the health emergency continued, it became impractical to categorize positive patient samples based on N gene Ct values due to the high workload. To expedite the sequencing process, we optimized our workflow with a standardized PCR cycle number for library amplification, allowing us to batch-process library amplification and reduce the total time required for library preparation. In total, the optimized process, bypassing qPCR viral copy estimation based on N gene Ct value, has reduced protocol time by approximately 2–3 h compared to the standard Ion AmpliSeq SARS-CoV-2 insight research assay protocol. Workflow optimization was supported by the evaluation of prior runs that involved N gene Ct data and assessing corresponding NGS data quality.

Research labs have developed open-source bioinformatic tools and workflow pipelines to analyze and report SARS-CoV-2 results. For instance, tools such as ViralFlow and CoVEx were developed in Python-3.8 [29,30]. They support the analysis and visualization of SARS-CoV-2 variant sequences obtained using Illumina sequencing technology. ViralFlow supports input files that are paired-end sequencing and CoVEx accepts both paired-end and single-end as input files. Multiple other tools were developed and tailored to address general challenges, lab-specific goals, sequencing techniques, or as an enhancement to existing pipelines [31].

There are three broad components of surveillance workflows: (1) genomic sequencing, (2) compiling and analyzing data, and (3) publishing findings to central repositories. To that end, we developed an analysis pipeline using Workflow Description Language (WDL), originally developed by the Broad Institute for their genomic analysis needs and now available as OpenWDL [32]. To develop an analysis pipeline which is sequencing platform agnostic, our workflow uses a consensus FASTA file as input. The final output files at the end of the run are integrated back into REDCap to form a central repository from which data can be pulled for further analysis, visualization and to generate outputs that fit the submission requirements for data repositories like GISAD and the Centers for Disease Control.

## 2. Materials and Methods

### 2.1. Specimens

Swab specimens are sourced from nasopharyngeal, oropharyngeal, or bronchoalveolar lavage (BAL) at OSU Wexner Medical Center. A unique sample ID and biorepository library ID are used to track each sample in the sequencing workflow and data analysis pipeline.

### 2.2. Capturing Data in REDCap Forms

Data is collected and managed using REDCap (version 17.0.8) (Research Electronic Data Capture) tools hosted at The Ohio State University College of Medicine [33,34]. Workflow-specific REDCap forms are used to capture data from receipt of the sample and initiation of the run to the conclusive report generation. These RedCap forms are designed to collect and validate data associated with sequencing runs. It includes anonymized patient and sample information, run-specific details (date, time, technician name), reagent lot numbers, analysis dates, QC data, bioinformatics analysis dates and report submission timelines. This structured approach ensured comprehensive documentation of all procedural and operational aspects of our sequencing workflow.

### 2.3. RNA Extraction, cDNA Synthesis

Total viral RNA was extracted using the KingFisher (Thermo Fisher Scientific, Waltham, MA, USA) automated nucleic acid purification system (cat. A42352) following the manufacturer’s recommended protocol. RNA was then reverse transcribed into stable cDNA using the IonTorrent™ NGS Reverse Transcription kit (cat. A45003; Thermo Fisher Scientific, Waltham, MA, USA) for NGS library preparation. Briefly, 7 µL of extracted RNA was added to each well containing 3 µL of the kit’s master mix on a PCR plate. Equal reaction volumes were used for negative and positive controls: nuclease-free water for the negative control and 7 µL (approximately 96 viral copies per µL) of diluted, non-infectious Twist Synthetic SARS-CoV-2 RNA Control14 (B.1.1.7_710528) (cat. NC1954840; Twist Biosciences, San Francisco, CA, USA) for the positive control.

### 2.4. Target Amplification, Digest, and Adapter Ligation

To prepare amplicon libraries we used the Ion Ampliseq Library Kit Plus (cat. A38875; Thermo Fisher Scientific, Waltham, MA, USA) which targets 237 amplicons of SARS-CoV-2 even with low-concentrated cDNA samples. Following kit protocol, 4.5 µL of Ion AmpliSeq HiFi mix and 3.5 µL of nuclease-free water were combined for a total volume of 8 µL and then added to each cDNA well from the previous step. Each sample well was then split into two pools, where 2 µL of both forward and reverse Ion AmpliSeq primers were added to the respective pool.

Given the significant increase in samples and the urgent need for testing during a global pandemic, we deviated from the standard Ion Ampliseq protocol recommending different cycle numbers based on viral load. All cDNA samples were amplified with AmpliSeq primer pools at 17 amplification cycles on the thermocycler (Thermo Fisher Scientific, Waltham, MA, USA) as described in the Ion Ampliseq protocol. This enabled us to efficiently process a higher volume of samples with reduced reagent consumption and faster turnaround times without compromising on accuracy.

Post-amplification, pools were combined and treated with 2 µL FuPa reagent for partial digestion, followed by a standard digestion cycle on the thermocycler. Finally, the digested amplicons were ligated to Ion Xpress Barcode Adapters following the manufacturer-recommended ligation cycle.

### 2.5. Library Purification and Library Quantification

Purification of the library was performed by the magnetic bead approach using High Prep PCR Clean Up (cat. AC-60250; MagBio Genomics Inc., Gaithersburg, MD, USA). Briefly, 36 μL of the High Prep PCR reagent was added to each sample well at the end of the ligation cycle with thorough mixing and incubation on a magnetic rack, followed by two washes with 160 μL of 80% ethanol each. The final pellet was resuspended using 50 μL TE buffer. A 1:100 sample dilution is performed at this step for sample library quantification using the Ion Library TaqMan^®^ Quantitation Kit (cat. 4468802, Thermo Fisher Scientific, Waltham, MA, USA). The standards for the Library TaqMan Quantitation kit (Thermo Fisher Scientific, Waltham, MA, USA) were prepared by prepping 5 serial dilutions of *E. coli* DH10B Control Library, in accordance with the user guide. After calculating equimolar amounts across the samples based on the quantification results, the sample libraries were pooled to get a total volume of 60 μL of an equalized sample library.

### 2.6. Ion Ampliseq Library Preparation and Ion S5 Sequencing

The Ion Chef instrument and the Ion Chip 540™ kit-chef reagents and solutions (catalog number A30011; Thermo Fisher Scientific, Waltham, MA, USA) were used for the preparation of sequencing libraries and templates. Starting with a 60 μL equalized sample library, 12.5 μL was diluted with 12.5 μL of TE buffer. This mixture was then transferred to a 200 μL microcentrifuge tube and loaded into the Ion Chef instrument for clonal amplification by mixing the libraries with Ion Sphere™ Particles (ISPs). The ISPs that were positive for the template were then enriched and automatically loaded onto the ion chip, ready for sequencing on the Ion GeneStudio S5.

### 2.7. SARS-CoV-2 Assembly and Consensus FASTA Data Generation

To analyze the quality of sequencing and generate the consensus FASTA file, Torrent Suite Software 5.12 was used with the default Ion AmpliSeq SARS-CoV2 insight reference sequence and .bed file provided. Further, command line versions of Pangolin and Nextclade were used for lineage and clade assignment.

### 2.8. WDL Workflow

To automate COVID-19 surveillance workflow and ensure reproducibility, scalability, and transparency, the WDL workflow was adopted. The workflow includes clade and lineage assignments, data transformation, and integration of viral mutations with patient demographic information. This pipeline leverages Nextclade and Pangolin command line tools and updates their reference files on every run, ensuring analyses reflect the latest genomic variations in the SARS-CoV-2 virus. Processed outputs from Nextclade and Pangolin were further refined using custom R scripts. These scripts were designed to integrate the lineage and clade outputs with quality control summaries from Ion Torrent sequencing runs. Upon processing, the data was then systematically uploaded to the REDCap database via API calls, eliminating the need for manual intervention as well as improving efficiency and accuracy.

### 2.9. COVID-19 Surveillance Dashboard

The COVID-19 Surveillance Dashboard is an interactive and dynamic tool developed using the [35] for R package, along with reactable [36], plotly [37], and other relevant dependency packages [38,39,40]. This dashboard leverages the Shiny modular programming approach, ensuring it is highly adaptable to other similar studies and can easily accommodate updates and modifications.

## 3. Results

### 3.1. Optimization of Sequencing Throughput and Quality Assessment

**Figure 1:**
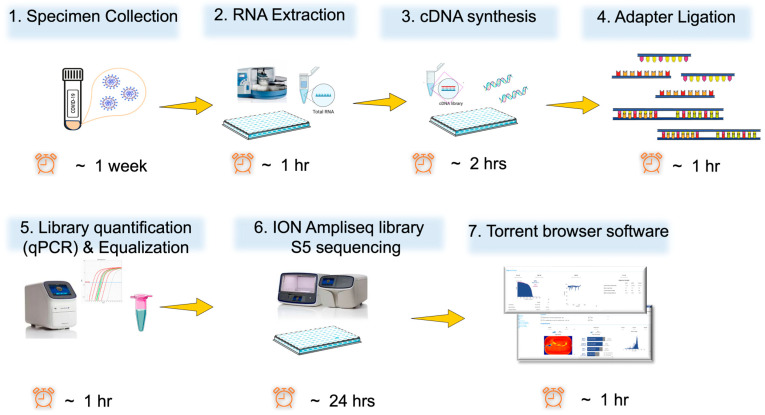
Schematic representation of the COVID-19 Ion Torrent protocol with approximate runtime. The process involves collection of a specimen, followed by RNA extraction. The extracted RNA is then reverse transcribed into cDNA; subsequently, adapter ligation and digestion are performed. Library quantification is performed using qPCR and equimolar amounts across samples are pooled to create equalized sample library. The prepared sample undergoes Ion AmpliSeq library preparation and sequencing using S5. Finally, the data is reviewed using the Torrent browser software 5.18.

**Figure 2:**
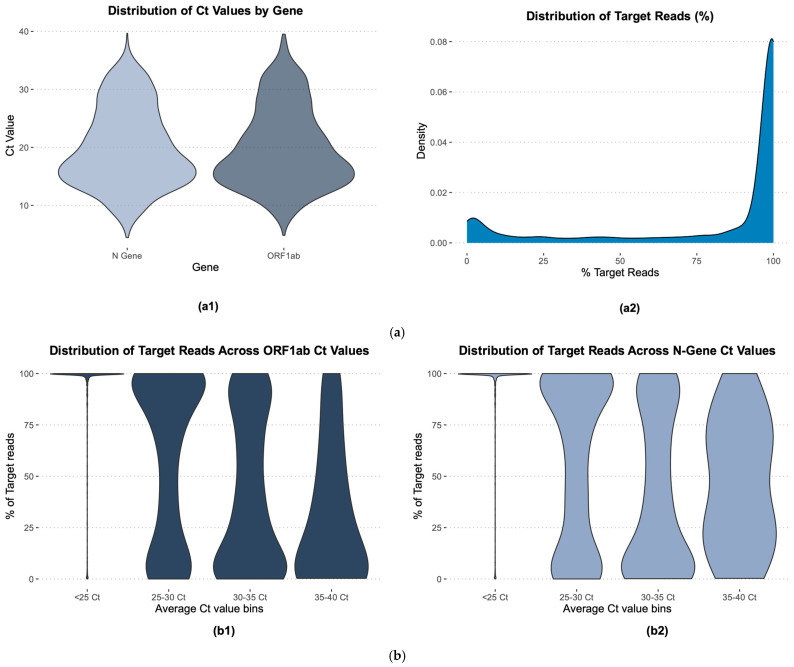
(**a**) Run quality assessment plots. (**a1**) Violin plots of Ct values for ORF1ab and N Gene, indicating viral load across samples (*n* = 2456). (**a2**) A density plot showing the distribution of target reads as a percentage of mapped reads, where a tight distribution around a high value suggests efficient target capture and sequencing. (**b**) Distribution of target sequencing reads mapped to the ORF1 and N gene regions. Violin plot illustrating the distribution of detected target reads across different Ct ranges of the respective (**b1**) ORF1ab and (**b2**) N genes. Maximum target reads achieved around Ct < 25 indicating the accuracy and reliability of the results.

The schematic representation of the COVID-19 Ion Torrent protocol in Figure 1 outlines a comprehensive workflow, including approximate runtimes for each step. The entire process, from library preparation to sequencing, is designed to be efficient and reproducible. However, patient samples exhibit high variability in the extraction process and the amount of RNA/cDNA generated for the respective sample varies. This variability can impact the consistency and quality of sequencing results. We initially categorized samples into low or high amplification groups based on viral copy numbers derived from N gene Ct values using TaqPath™ COVID-19 Combo Kits. However, as the sample load increased, it became impractical to run each sample individually and batch them based on N gene Ct values. To ensure the reliability of our surveillance studies, we conducted quality assessment plots on (*n* = 2456) [Figure 2a,b], using ORF1ab and N genes as control targets for our optimization studies. As shown in Figure 2a, post-sequencing data analysis was performed to review sequencing output quality. A density plot illustrating the distribution of target reads as a percentage of mapped reads shows a narrow distribution centered around a low Ct value, indicating efficient target capture and sequencing. Additionally, we investigated the distribution of the percentage of reads detected for different ranges of Ct values. A strong negative correlation was observed between Ct values and the percentage of target reads for both the N gene (Spearman ρ = −0.60, *p* < 2.2 × 10^−16^) and ORF1ab (Spearman ρ = −0.61, *p* < 2.2 × 10^−16^). Specifically, a higher number of target reads, approaching 100%, is observed at lower Ct values, while higher Ct values exhibit greater variability in read distribution. This observation is consistent with prior studies demonstrating an inverse relationship between Ct values and viral load [41]. Although substantial variability is observed at higher Ct values, which is biologically expected in bulk sequencing, the consistency of this relationship across both gene targets supports the robustness of Ct as a proxy for sequencing performance in our runs.

Leveraging this observation, we have followed recommended amplification cycles (17 cycles) in ion ampliseq protocol for high-load suspected samples that have consistently produced sufficient cDNA when batching was carried out based on N gene Ct values. Based on this finding, we empirically determined the optimal amplification cycle number as 17 to amplify cDNA targets for all samples eliminating the need for a step involving qPCR quantification of the N gene. This adjustment not only streamlined the turnaround time but also maintained data integrity across samples.

### 3.2. REDCap Enhances the Workflow Management and Accessibility in Sequencing Workflows

REDCap provides an intuitive interface for validated data capture, audit trails, and tools for data integration and interoperability with external sources. One new and advanced module of the REDCap is Clinical Data Interoperability Services (CDIS) released in 2021 and based HL7 standards on FHIR [42]. This module allows individual REDCap projects to interact with an electronic health record (EHR) such as EPIC and Cerner and to pull selected information from the EHR into the REDCap project [34]. While the CDIS module would seamlessly integrate with EHR, our current approach ensures that our REDCap forms and data processes can adapt to the CDIS feature once it becomes available. In contrast to the primary intended use of REDCap, which is designed for survey data collection, we utilized it as a storage and workflow management solution for our surveillance efforts. Our decision to adopt REDCap over setting up a dedicated database server was driven by its wide use in clinical and research groups, secure API tools and user-friendly interface. Moreover, it avoids the complexities that a dedicated database server, which necessitates additional investment in IT support. Thus, we processed the protected patient data files from Epic via R scripts and automated the anonymized data load via an API token provided by REDCap. Similarly, we also captured the sequencing run information and the analysis results as separate projects under REDCap. The significance of this data integration into the REDcap databases has ensured data security and integrity, facilitated seamless workflow management, and enhanced data accessibility.

### 3.3. WDL Enhances Automation and Integration Different Analysis Pipeline Steps

**Figure 3:**
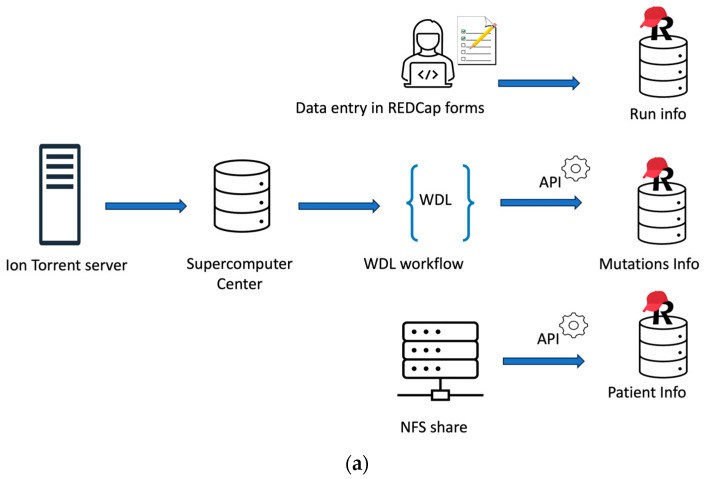

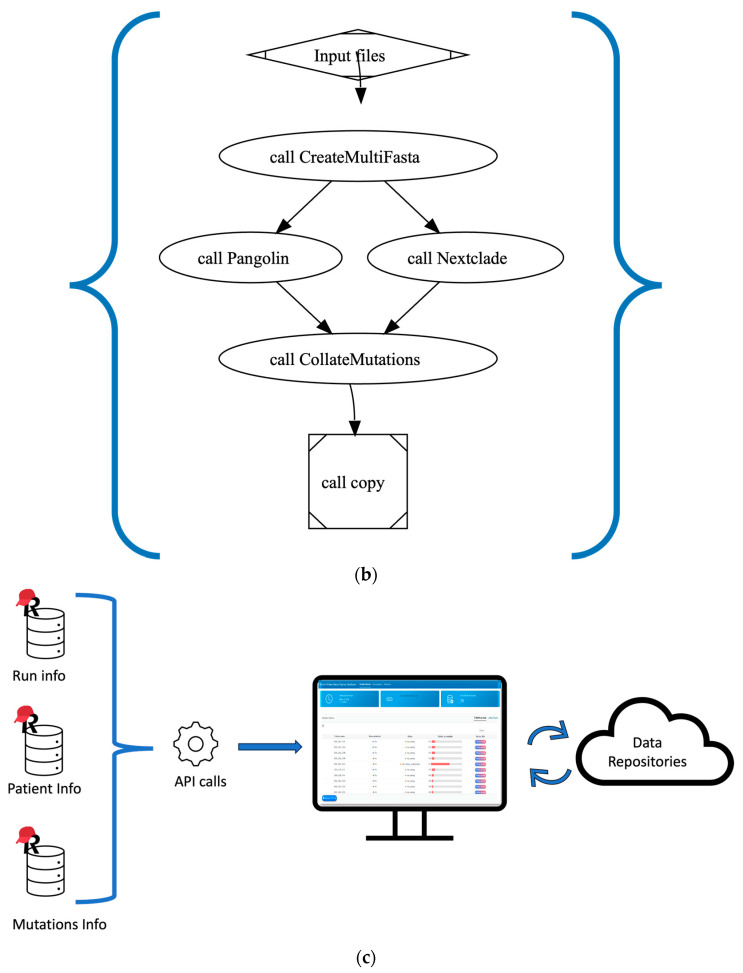
(**a**) Data integration, management, and analysis. The three-step process of data integration in our REDCap system. *Top*: A technician manually enters run information into customized REDCap forms which is stored in Run info database. *Middle*: The WDL pipeline to process raw data, integrate QC info and identify mutations. The output data is eventually stored into mutation info database in REDCap via API calls. *Bottom*: Patient information is retrieved from Excel files stored on an NFS share and stored into the Patient Info database in REDCap via API calls. (**b**) WDL workflow: Illustrates the detailed WDL steps, which begins with input files and includes steps invoking respective program files to create required outputs, collating mutations, and copying results to mutation info database. This pipeline is triggered weekly to process raw data. (**c**) Complete data analysis pipeline: This showcases the integration of three REDCap databases (run information, patient information, mutation data) accessed via API calls into an R Shiny data portal for visualization, analytics and report export to data repositories. This pipeline is triggered by the user clicking the refresh API button on the dashboard.

Workflow Management Systems such as Workflow Description Language (WDL), Nextflow, and Snakemake offer an efficient and systematic way to build genomic analysis pipelines that handle large volumes of data and require different programming languages to process data. The choice of a workflow management system depends on specific requirements of the project, the computational environment, and the user’s familiarity with the underlying languages [42]. We chose WDL for its human-readable and writable syntax and its easy setup with quick execution using the Cromwell engine (Cromwell v88). Figure 3a captures the overall data analysis and data management pipeline. We democratized our WDL pipeline (Figure 3b) for non-informatics users with a single command to run which circumvents the need for manually editing configurations in JSON format. Furthermore, we have implemented an automated cron job that monitors the input folder for new data and ensures the WDL pipeline is executed promptly. This automation not only processes the input files but also integrates the resulting mutation and variant data directly into the REDCap database (Figure 3c), thereby eliminating reliance on specialized personnel and enhancing the robustness of our workflow.

### 3.4. Interactive Dashboard Empowers Users with Visualization for In-Depth Exploration

As we advance in genomic research, interactive dashboards are not merely a technological advancement: they offer a transformative approach to managing complex genomic data. We could successfully leverage the available open-source tools and provide integrated data to dynamically display our analysis pipeline. Interactive plots and tables offered a visual means to monitor laboratory runs and track the identified mutations, lineages, and clades over time. Moreover, the ability to integrate the different data sources has streamlined the generation of a comprehensive variant report. The dashboard not only aides as a source to monitor our workflow and results, but it also provides a consolidated variant report adhering to the submission criteria mandated by public repositories, such as GISAD and the Ohio Department of Health.

## 4. Discussion

The COVID-19 pandemic has underscored the importance of surveillance programs for global public health security. The unprecedented scale of the pandemic necessitated a coordinated response that leveraged scientific evidence to inform strategic decision-making by governments worldwide. Effective surveillance studies have been the cornerstone of this response, enabling real-time tracking of the virus’s spread, identification of emerging viral variants, and identifying hot spots. The SARS-CoV-2 virus presents a unique disease dynamic in an ever-changing genomic landscape. The rapid introduction of advanced sequencing approaches and bioinformatics tools provided a foundation for laboratories across the world to adapt swiftly and sequence SARS-CoV-2 from infected patients. As part of the adaptation, our laboratory had to handle a rapid increase in sample volume, which led to an optimization of workflow efficiency while still yielding good data. As a result, this streamlined workflow process can be implemented by any laboratory and expanded upon.

The synergistic progress of global surveillance and research laboratories has culminated in the creation of extensive data repositories, such as GISAID, which have become instrumental in advancing our understanding of infectious diseases. These repositories serve as a nexus for knowledge dissemination, catalyzing a collaborative research environment that spans across various domains, including epidemiological studies, vaccine development, and public health policy.

As we reflect on the COVID-19 pandemic, it becomes clear that robust surveillance workflows are vital to fight against emerging infectious diseases. It is imperative that these workflows remain robust, adaptable, and responsive to the ever-changing landscape of global health threats. While there are several frameworks for SARS-CoV-2 genomic surveillance such as ARTIC-based sequencing protocols [43] and user-friendly web-based bioinformatics platforms for clade assignment such as Nextstrain [25] for clade assignment and phylogenetic visualization, our approach does not aim to introduce a fundamentally new sequencing approach or bioinformatics workflow pipeline. Instead, it focuses on workflow integration and operational efficiency. Although numerous dashboards have been developed for SARS-CoV-2 genomic surveillance [25,28,44], the focus of these dashboard designs is for downstream visualization of genomic and epidemiological trends rather than real-time monitoring of laboratory workflows. A recent commentary on the importance of pathogen genomic surveillance in healthcare has identified certain barriers that prevent early detection of outbreaks. These include cost, expertise, and lack of standardized methodologies and incentives [45]. It highlights the need for strategic investment and cross-sector collaboration. Our system extends beyond existing approaches by integrating data capture (via REDCap), sequencing analysis, and interactive reporting within a single dashboard. This integrated design reduces reliance on manual data consolidation and directly addresses challenges associated with siloed surveillance pipelines. Moreover, this framework offers a cost-effective and standardized solution while reducing the technical expertise required through an end-to-end integrated pipeline.

Our attempts to modularize and streamline the workflow process is a step towards creating an easily adaptable analysis workflow for new or existing surveillance laboratories. By integrating REDcap within this process, our laboratory was able to track instrumentation, lot numbers, laboratory technicians, and protocol steps in real time. Capturing such information is critical for troubleshooting and this enables any clinical laboratory to comply with CAP/CLIA regulations in regard to documentation when adopting this pipeline. Further, the findings of this study suggest that the modified protocol streamlined laboratory operations while maintaining high precision and faster processing of the samples. This makes it a promising approach for laboratories to adopt, particularly those with high throughput sample flow. As the number of patient samples grew, we realized the advantages of having a centralized system to integrate and monitor data in real time. Our interactive analysis dashboard helped us efficiently monitor laboratory protocol steps as well as generate surveillance reports for submission to repositories. Briefly, our system provides a detailed, localized view of our surveillance laboratory outcomes.

While our overall process pipeline and dashboard streamlines the workflow which was often in silos and addresses challenges in monitoring and consolidating the results, we must also acknowledge its limitations. The workflow is tested and implemented specifically to data generated from SARS-CoV-2 sequencing using S5 Ion torrent and may present challenges for direct adaptation in other clinical laboratories. These challenges are pertained to the specific structure of the data files and sequence of analysis. However, the modular nature of our approach allows customizations and adaptations to other sequencing platforms or epidemiological studies. We believe that the workflow infrastructure we have developed is not only relevant for the SARS-CoV-2 virus but also serves as a blueprint for future surveillance studies for a wide array of infectious diseases.

This study focuses on workflow engineering and automation. Its modular architecture provides a strong foundation for future analytical extensions. The ability to integrate heterogeneous data sources into a unified system enables opportunities for more advanced downstream analyses.

## 5. Conclusions

Our approach represents a significant advancement in SARS-CoV-2 genomic surveillance. By combining REDCap, interactive dashboards, and open-source tools, we offer a secure solution for the timely tracking of viral variants. In addition, we optimize library amplification by running all samples on the same PCR without compromising the accuracy of results. This workflow has been successfully implemented in our institution and is designed to be easily adopted by other laboratories. REDcap and the interactive dashboards are user friendly, allowing laboratory technicians to move through the workflow with ease. The use of secure platforms like REDcap ensures the confidentiality and integrity of patient data. Our approach is easy to implement and designed to be adaptable and scalable, allowing for increased sample throughput as needed. Such flexibility is important for widespread adoption and implementation. It is also adaptable to other surveillance programs utilizing bench-top ion torrent sequencer and open-source tools for automation. The use of open-source tools enhances the reproducibility and transparency of the workflow, which is essential for scientific collaboration. The interactive dashboards provide comprehensive information and allow users to explore workflow progress, genetic variants, and assess temporal trends. Overall, this automated workflow offers a step forward in epidemiological investigations.

## Data Availability

The demo version of the shiny dashboard can be accessed at https://gadepallivs.shinyapps.io/covid19_survelliance_demo_app/? (accessed on 26 May 2026). The code implemented for this project, WDL pipeline, R/Shiny Interactive dashboard is hosted on the institutional GitLab repository https://code.osu.edu/gadepalli.3/covid_surveillance_demo/-/tree/main (accessed on 26 May 2026).

## Abbreviations

The following abbreviations are used in this manuscript:

GISAD	Global Initiative on Sharing All Influenza Data
REDCap	Research Electronic Data Capture
WDL	Workflow Description Language
COVID-19	Coronavirus disease 2019
SARS-CoV-2	Severe acute respiratory syndrome coronavirus 2
